# Tumeurs du cône médullaire et de la queue de cheval associées à l’hydrocéphalie chronique: à propos de 2 cas: Tumors of the conus medullaris and of the cauda equina associated with chronic hydrocephalus: about 2 cases

**DOI:** 10.11604/pamj.2018.29.206.10723

**Published:** 2018-04-09

**Authors:** Mohamed Khoulali, Mohammed Yasssine Haouas, Jihad Mortada, Robin Srour

**Affiliations:** 1 Université Mohammed Premier, Faculté de Médecine et de Pharmacie, CHU Mohamed VI, Oujda, Maroc,; 2 Université Hassan 2, CHU Ibn Rochd, Casablanca, Maroc,; 3 Hôpital Louis Pasteur, Colmar, France

**Keywords:** Tumeurs médullaires, hydrocéphalie chronique, hyperprtéinorachie, chirurgie, Medullary tumors, chronic hydrocephalus, high cerebrospinal fluid protein, surgery

## Abstract

L’hydrocéphalie chronique associée à une tumeur du cône médullaire et /ou de la queue de cheval est extrêmement rare. Nous présentons deux patients porteurs d’une tumeur médullaire révélée par la triade: démence, troubles de la marche et incontinence urinaire. L’imagerie par résonance magnétique (IRM) cérébrospinale objectivait une hydrocéphalie communicante et une tumeur intradurale siégeant au niveau du cône médullaire et de la queue de cheval. L’exérèse chirurgicale d’un schwannome bénin et d’un épendymome a permis la résolution de la symptomatologie clinique due à l’hydrocéphalie sans procéder à un shunt ventriculaire. Une dizaine de cas de démence et d’hydrocéphalie accompagnant une tumeur spinale sont rapportées. Une variété de mécanismes a été proposée pour expliquer cette association mais la physiopathologie exacte reste mal connue.

## Abstract (en)

*Chronic hydrocephalus associated with a tumor of the conus medullaris and/or of the cauda equina is extremely rare. We here report two cases of medullary tumor revealed by the triad: dementia, difficulty walking and urinary incontinence. Magnetic resonance imaging (MRI) of the cerebrospinal fluid showed communicating hydrocephalus and intradural spinal tumors at the level of the conus medullaris and of the cauda equina. Surgical resection of a benign schwannoma and of an ependymoma allowed the resolution of the clinical symptomatology due to hydrocephalus without implantation of ventricular shunt. A dozen cases of dementia and hydrocephalus associated to spinal tumor have been reported. A variety of approaches have been proposed to explain this association but the exact pathophysiology is not accurately known.*

## Introduction

L’hydrocéphalie chronique de l’adulte est décrite pour la première fois par Adams et Hakim en 1965. Elle est le plus souvent tributaire à une hémorragie méningée, un traumatisme ou une méningite. Elle est rarement associée à une tumeur spinale. Nous présentons deux patients, un avec un schwannome du cône médullaire et l’autre avec un épendymome de la queue de cheval, associés à un tableau de l’hydrocéphalie chronique et chez qui les symptômes sont complètement résolus après l’exérèse tumorale. Nous présentons les détails de ces deux cas et nous discutons les mécanismes et la prise en charge de l’hydrocéphalie secondaire à la tumeur spinale.

## Patient et observation

### Cas 1

**Figure 1:**
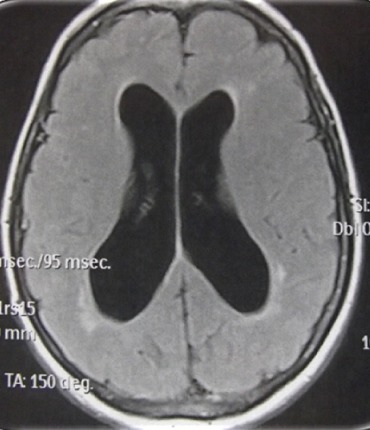
Cas1/ IRM cérébrale en coupe axiale séquence Flair, montrant une dilatation ventriculaire avec signe de résorption transépendymaire, sans signes de métastases leptoméningée

**Figure 2:**
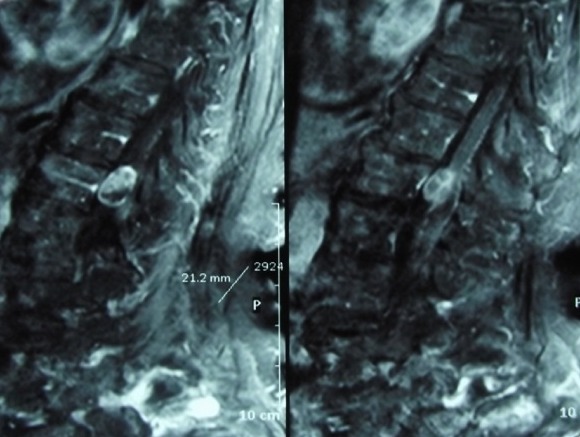
Cas 1/ IRM de la charnière dorsolombaire en coupe sagittale, séquence T1 après injection du gadolinium, montrant une lésion intradurale extramédullaire siégeant en regard de la deuxième vertèbre dorsale (D12) paramédiane gauche et qui se rehausse de façon intense, évoquant une tumeur du cône médullaire

**Figure 3:**
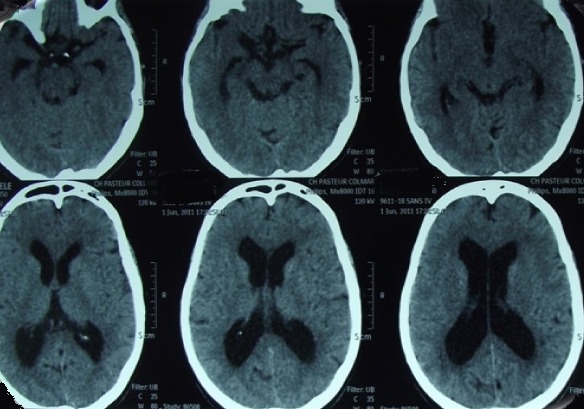
Cas 1/scanner cérébral de contrôle réalisé à 6 mois en post opératoire, montrant une diminution de la taille ventriculaire et de l’hydrocéphalie

Madame K.L. âgée de 75 ans, autonome, ayant comme antécédents une artériopathie carotidienne droite et opérée pour un goitre thyroïdien et substituée par Lévothyrox 75 mcg/ jour. En septembre 2010, elle a été victime d’une Chute occasionnant chez elle un traumatisme crânien bénin sans perte de connaissance, restée sans suites. Et depuis les chutes se succèdent, un scanner cérébral a été réalisé qui a montré une hydrocéphalie tétraventriculaire avec signes de résorption transépendymaire, le diagnostic de l’hydrocéphalie chronique a été suspectée, une ponction lombaire déplétive et analyse de liquide cérébrospinal était en faveur d’une hyper-protéinorachie à 3,23 g /dl et la pression était de 14cmH2O. Elle se présentait début janvier 2011 avec une confusion et désorientation temporo-spatiale, une marche extrêmement difficile nécessitant l’aide de deux personnes et des troubles mnésiques majeurs reconnaissant à peine ses enfants. La patiente rapportait également la notion des dorso-lombalgies avec irradiation dans le membre inferieur gauche. L’examen clinique n’objectivait pas de déficit moteur, par contre il existait un signe de Babinski et le reflexe rotulien était vifs au membre inferieur gauche. Toute cette symptomatologie nous a fait d’emblée évoquer la possibilité d’une tumeur médullaire responsable d’hydrocéphalie. Une IRM cérébrospinale en pondération T1, T2 et T1 avec injection de gadolinium montrait à l’étage encéphalique une hydrocéphalie communicante avec résorption trans-épendymaire sans signes demétastases leptoméningées (Figure 1). À l’étage médullaire, il existaitune lésion intra durale extra médullaire siégeant en regard de la vertèbre D12 paramédiane gauche responsable d’une importante compression médullaire. La lésion est spontanément hypointense T1 et hyperintense T2 et qui se rehausse de façon intense et hétérogène (Figure 2). Nous avons décidé d’intervenir sur la lésion médullaire dont le niveau lésionnel nous a incité à compléter par une artériographie médullaire, cela nous a permis de repérer l’artère d’Adamkiewicz qui naissait en D12 gauche . L’intervention chirurgicale a permis une exérèse complète. L’examen anatomopathologique montrait une prolifération tumorale à cellules fusiformes sans signes de malignité évoquant un schwannome. L’évolution a été lentement favorable chez cette patiente qu’on a pu recommencé à mobiliser progressivement, la mise au fauteuil a pu se faire rapidement sans problème puis la marche a pu se faire d’abord avec deux aides, puis progressivement avec un déambulateur et l’aide d’un kinésithérapeute. Sur le plan cognitif on a constaté assez rapidement une amélioration de la vigilance et une amélioration lente des troubles mnésiques avec disparition complète au bout de 6 mois d’évolution. La protéinorachie a nettement baissé à 0,29g/L et le scanner cérébral de contrôle réalisé à 6 mois montrait une régression de la dilatation ventriculaire (Figure 3).

### Cas 2

**Figure 4:**
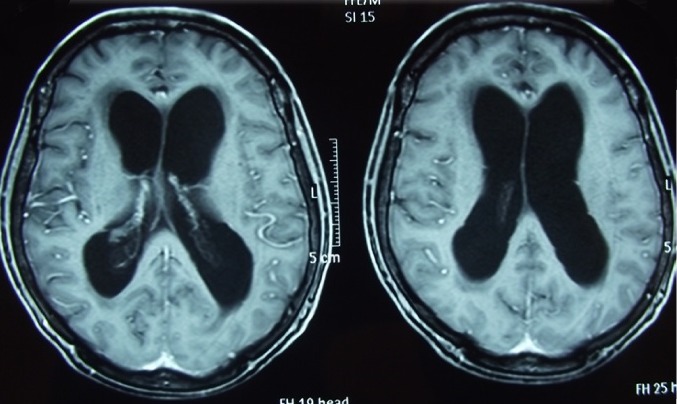
Cas 2/ IRM cérébrale en coupe axiale, séquence T1 avec injection du gadolinium, montrant une hydrocéphalie avec signes de résorption transépendymaire, sans atteinte leptoméningée ou intraparenchymateuse

**Figure 5:**
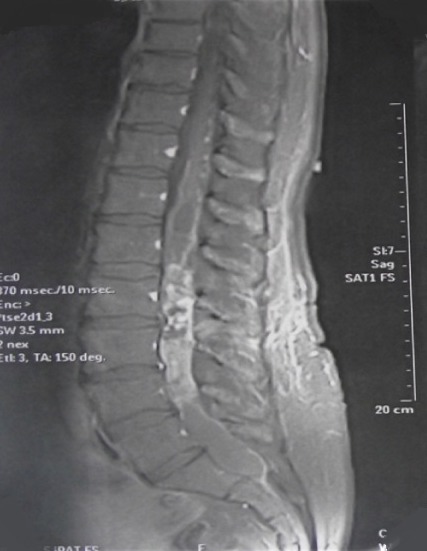
Cas 2/ IRM lombaire en coupe sagittale, en séquence T1 avec injection du gadolinium, montrant lésion siégeant au niveau de la queue de cheval qui se rehausse de façon hétérogène après injection du gadolinium

**Figure 6:**
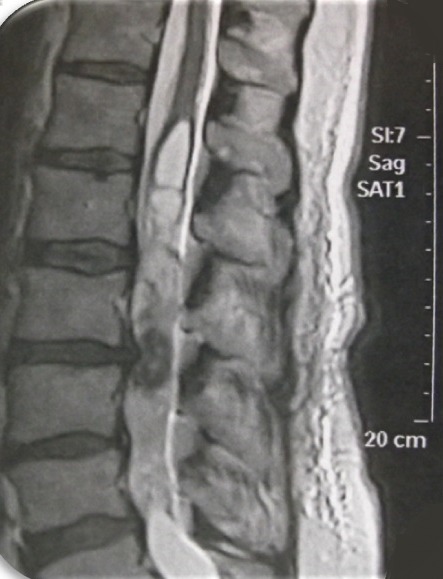
Cas 2/ IRM lombaire en coupe sagittale, en séquence T2, montrant lésion siégeant au niveau de la queue de cheval associée à un ramollissement hémorragique

**Figure 7:**
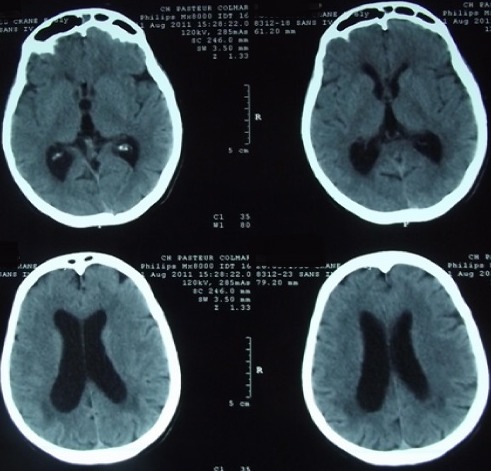
Cas 2/scanner cérébral de contrôle réalisé à 3 mois en post opératoire, montrant une diminution de la taille ventriculaire et de l’hydrocéphalie

Madame K.D, âgée de 61 ans, elle est suivie pour une dépression majeure depuis 2 ans puis la symptomatologie est marquée par la survenue progressive sur 3 mois des troubles mnésiques, des troubles de la marche et de l’équilibre et des troubles sphinctériens à type d’incontinence urinaire nocturne. Le reste de l’examen clinique était sans particularité. Un scanner cérébral a été réalisé qui montrait une hydrocéphalie communicante. Une ponction lombaire était hémorragique et non concluante. Une aggravation neurologique rapidement progressive sur 3 jours a été observée, la patiente est devenue grabataire. L’examen clinique objectivait une paraparésie avec abolition des réflexes ostéotendineux des 2 membres inferieurs. L’IRM cérébrospinale montrait,à l’étage cérébral une hydrocéphalie tétraventriculaire avec résorption transépendymaire sans signes de métastases leptoméningées (Figure 4). À l’étage médullaire, elle montrait une volumineuse tumeur étendue du cône médullaire et de la queue de cheval avec ramollissement hémorragique (Figure 5 et Figure 6). La patiente a bénéficié d’une angiographie médullaire qui a permis de repérer l’artère d’Adamkiewicz au niveau de D10 c'est-à-dire au pôle supérieur de la tumeur. Une laminectomie de D11 à L5 est réalisée avec exérèse macroscopiquement complète de la tumeur. L’examen anatomopathologique était en faveur d’un épendymome grade 2. La patiente a été adressée dans un centre spécialisé en rééducation fonctionnelle. L’évolution était favorable pour cette patiente qui était grabataire en préopératoire avec régression quasi-complète des troubles de la mémoire avec persistance des troubles moteurs et sphinctériens mais qui ont tendance à s’améliorer progressivement. Un scanner cérébral réalisé à 3 mois objectivait une diminution de la taille ventriculaire (Figure 7).

## Discussion

Le développement de l'hydrocéphalie secondaire à une tumeur spinale thoracolombaire a été rapporté pour la première fois par Kyrielieis en 1931. Et depuis environ 300 cas de tumeurs spinales associées à une hydrocéphalie et l’hypertension intracrânienne sont rapportés dans la littérature. Par ailleurs il y’a très peu de cas de tumeurs spinales dans lesquelles les troubles cognitifs constituaient le maitre symptôme. Le phénomène a d'abord été rapporté par Neil-Dwyer en 1973, une dizaine de cas supplémentaires ont été rapportées à ce jour [1]. Tous les patients sauf un étaient d'âge moyen ou âgées, et il n'y avait pas de différence de sexe. En plus d’une démence, les troubles de la marche sont présentés chez 6 cas et l'incontinence urinaire chez 4 patients. Il est à noter que cette triade clinique ressemble à celle de l’hydrocéphalie chronique de l’adulte. Dans tous les 10 cas dans lesquels une ponction lombaire a été réalisée, la protéinorachie a dépassé 500 mg / dl. Les diagnostics histologiques étaient un neurinome dans 5 cas, un neurofibrome dans 3 cas, un épendymome dans 2 cas et un cas d’oligodendrogliome. Certains épendymomes sont diagnostiqués précocement, révélés par la survenue brutale d’un tableau clinque périphérique lié à l’hémorragie sous arachnoïdienne d’origine tumorale. Alors que plusieurs patients ne se plaignent pas de symptomatologie médullaire et la tumeur n’est révélé qu’après le diagnostic de l’hydrocéphalie, la mise en place du shunt ventriculaire et la résolution des troubles mnésique. Par ailleurs les schwannomes produisent un tableau d’hydrocéphalie progressive et éventuellement des troubles mnésiques.

Une variété de mécanismes a été proposée pour expliquer l’association d’hydrocéphalie avec une tumeur spinale. Plusieurs auteurs relient l’hydrocéphalie à l’hyperprotéinorachie rencontrée fréquemment chez les patients atteints de tumeurs spinales [2]. L’augmentation de la viscosité et l’élévation soutenue de la résistance à l'écoulement du liquide cérébrospinal (LCS) semble plus probablement causée par la présence anormale du fibrinogène dans le LCS et sa conversion en fibrine dans l'espace sous-arachnoïdien et les villosités de Pacchioni. La présence anormale de cette protéine dans le LCS pourrait être l'effet d’une réaction inflammatoire chronique due à l'existence d'une lésion de la moelle, la rupture de la barrière hémato-encéphalique avec passage direct des protéines de fibrinogène et de sérum à travers la paroi pathologique des vaisseaux tumoraux, comme le confirment les tests de perfusion dans le cas de Borgesen et al. [3]. Le rôle important joué par la concentration anormale du fibrinogène dans LCS est fortement soutenue par plusieurs études : d’abord Le LCS prélevé au niveau lombaire ou cisternal ne montre pas seulement une hyperprtéinorachie, mais peut aussi coaguler spontanément dans le tube d'examen (Froin's syndrome), indiquant ainsi l'existence des taux élevés de fibrinogène dans certains cas de tumeurs de la moelle. Ensuite dans certains tumeurs intramédullaire, en particulier les épendymomes, l’hydrocéphalie est le résultat des saignements répétés de la tumeur dans l’espace méningé (syndrome de Fincher), ressemblant à la physiopathologie de l'hydrocéphalie secondaire à l’hémorragie sous-arachnoïdienne intracrânienne. En plus Brinker et al. ont démontré que la perfusion intrathécale de l'activateur du plasminogène tissulaire (Rt-PA), qui est une substance fibrinolytique, pourrait être efficace pour traiter l'augmentation de la résistance à l'écoulement de LCS et l'hydrocéphalie subaiguë observée après hémorragie sous-arachnoïdienne expérimentale [4]. Enfin la circulation lente du LCS dans les espaces méningés favoriseraient le dépôt et la conversion du fibrinogène en fibrine au niveau des espaces sous-arachnoïdiens de la convexité et / ou des citernes basales, induisant une arachnoïdite, des adhérences fibreuses et oblitération des espaces sous arachnoïdiens.

Morandi et al. plaident en faveur de la théorie hydrodynamique. En effet, ils ont proposé que l'hydrocéphalie chez les patients porteurs des tumeurs bénignes intrarachidiennes peut être causée par une réduction de la compliance du LCS secondaire à l’obstruction tumorale de l'espace sous-arachnoïdien [5]. Des études récentes par IRM sensible au débit soutiennent cette théorie [6]. Pendant la systole cardiaque, il y a une expansion de l'ensemble du volume intracrânien en raison de la transmission des pulsations artérielles cérébrales. Selon la doctrine Monro-Kellie l’augmentation systolique du volume intracrânien doit être compensée par la sortie du LCS du compartiment intracrânien vers les espaces sous-arachnoïdiens péri-médullaire. Les compartiments crâniens et intrarachidiens sont instantanément en équilibre et les fluctuations physiologiques de la pression du LCS sont rapidement tamponnées par l'espace sous-arachnoïdien rachidien et en particulier la partie lombaire du sac dural a été décrit comme un «réservoir élastique» pour l'écoulement de LCS [7]. Une obstruction peut isoler les espaces sous arachnoïdiens péri-médullaires du compartiment intracrânien et empêcher la distribution normale des variations de pression de LCS causant ainsi une hydrocéphalie et dilatation ventriculaire.

L’association d'une arachnoïdite néoplasique à l’hydrocéphalie a été signalée pour la première fois en 1975 par Maurice Williams et al. Chez 3 patients porteurs de tumeurs intramédullaires (deux schwannomes malins et un oligodendrogliome bénin). L'auteur a suggéré que la diffusion des cellules tumorales à travers les voies sous-arachnoïdiennes pourrait être responsable de l'hydrocéphalie [8].

Il semble licite, devant tout tableau d’hydrocéphalie mal expliqué, de réaliser directement une IRM cérébrospinale qui a l'avantage d'éviter tout geste agressif et potentiellement dangereux. Une dilatation tétra-ventriculaire avec signes de résorption transépendymaire sont fréquemment trouvées à l’imagerie. L’IRM offre un avantage clair en cas de tumeurs médullaire ou de la queue de cheval et une haute résolution pour détecter les métastases leptoméningées.

Si l’hydrocéphalie est diagnostiquée dans le même temps que la tumeur médullaire, l’idéal est d’éviter le shunt ventriculaire avant l’exérèse chirurgicale de la tumeur pour deux raisons principales: l’hydrocéphalie peut se résoudre spontanément après l’ablation de la tumeur en particulier pour les lésions extramédullaires et le risque de détérioration neurologique liée au shunt ventriculaire. En cas de gliome intramédullaire, l’IRM avec injection de produit de contraste doit être effectué pour éliminer une dissémination leptoméningée de la tumeur qui peut être l’origine de l’hydrocéphalie. Dans ce cas, l’hydrocéphalie est moins susceptible de disparaitre seulement par l’exérèse tumorale, une dérivation de LCS parait nécessaire si possible en même temps que l’intervention portant sur la tumeur. En outre, si l’hydrocéphalie apparait tardivement après l’exérèse de la tumeur, elle doit être considérée comme signe précoce de métastase intracrânienne, surtout en cas de tumeur gliale intramédullaire [9, 10].

## Conclusion

Depuis l’avènement de l’IRM, l’incidence des tumeurs médullaires associées à l’hydrocéphalie a été augmentée. En cas d’hydrocéphalie communicante, un examen neurologique et neuroradiologique minutieux à la recherche d’une tumeur médullaire est obligatoire, en particulier pour les hydrocéphalies inexpliquées, avant de procéder au shunt ventriculaire et à la ponction lombaire qui peuvent être dangereuses.

## Conflits d’intérêts

Les auteurs ne déclarent aucun conflit d'interêts.

## References

[1] Feldmann E, Bromfield E, Navia B (1986). Hydrocephalic dementia and spinal cord tumor. Report of a case and review of the literature. Arch Neurol.

[2] Harris P (1962). Chronic progressive communicating hydrocephalus due to protein transudates from brain and spinal tumours. Dev Med Child Neurol.

[3] Borgesen SE, Sorensen SC, Olesen J (1977). Spinal tumors associated with increased intracranial pressure - Report of two cases and a discussion on the pathophysiology. Acta Neurol Scand.

[4] Brinker T, Seifert V, Dietz H (1992). Subacute hydrocephalus after experimental subarachnoid hemorrhage: its prevention by intrathecal fibrinolysis with recombinant tissue plasminogen activator. Neurosurgery.

[5] Morandi X, Amlashi SF, Riffaud L (2006). A dynamic theory for hydrocephalus revealing benign intraspinal tumours: tumoural obstruction of the spinal subarachnoid space reduces total CSF compartment compliance. Med Hypotheses.

[6] Raksin PB, Alperin N, Sivaramakrishnan A, Surapeni S, Lichtor T (2003). Noninvasive intracranial compliance and pressure based on dynamic magnetic resonance imaging of blood flow and cerebrospinal fluid flow: review of principles, implementation and other noninvasive approaches. Neurosurg Focus.

[7] Mokri B (2001). The Monro-Kellie hypothesis - Applications in CSF volume depletion. Neurology.

[8] Maurice-Williams RS, Lucey JJ (1975). Raised intracranial pressure due to spinal tumors: 3 rare cases with a probable common mechanism. Br J Surg.

[9] Maroulis H, Maixner WJ, Leone E, Cinalli G, Cinalli G, Maixner WJ, Sainte-Rose C (2004). Hydrocephalus and spinal tumors. Pediatric hydrocephalus.

[10] Giuseppe Mirone, Giuseppe Cinalli, Pietro Spennato, Claudio Ruggiero, Ferdinando Aliberti (2011). Hydrocephalus and spinal cord tumors: a review. Child's Nervous System.

